# Impact of dyslipidemia on 24-h urine composition in adults without urolithiasis

**DOI:** 10.1186/s12944-018-0899-x

**Published:** 2018-11-06

**Authors:** Chao Cai, Zanlin Mai, Tuo Deng, Zhijian Zhao, Wei Zhu, Yaoan Wen, Xiaolu Duan, Wenqi Wu, Guohua Zeng

**Affiliations:** 1 Department of Urology, Minimally Invasive Surgery Center, The First Affiliated Hospital of Guangzhou Medical University, Guangzhou, China; 2 Guangzhou Institute of Urology, Guangzhou, China; 3 Guangdong Key Laboratory of Urology, Guangzhou, China

**Keywords:** Dyslipidemia, 24-h urine composition, Non-stone formers, Effects

## Abstract

**Purposes:**

To evaluate the influence of dyslipidemia on 24-h urine composition in adults who were non-stone formers (NF).

**Methods:**

Samples for 24-h urine composition were analyzed from 584 NF adults without urolithiasis in a national six-city-based epidemiologic study. The samples were divided into groups based on total cholesterol (TC), triglycerides (TG), high-density lipoprotein (HDL), and low-density lipoprotein (LDL). The groups were compared based on demographic data and each component of 24-h urinalysis.

**Results:**

The numbers of participants in high TG, high TC, high LDL, and low HDL were 106, 175, 147, and 59, respectively. The high TG group had increased urinary excretions of oxalate [mean difference (MD) = 0.032 mmol, 95% confidence interval (CI): 0.000–0.065] and potassium (MD = 4.298 mmol, 95%CI: 0.182–8.414). Increased urinary excretion of calcium (MD = 0.531 mmol, 95%CI: 0.061–1.001), sodium (MD = 41.561 mmol, 95%CI: 9.179–73.942), and chloride (MD = 45.209 mmol, 95%CI: 12.118–78.299) were found in the high TC group. Interestingly, the high LDL group had a decreased urinary excretion of calcium (MD = − 0.573 mmol, 95%CI: -1.048 to − 0.097), oxalate (MD = − 0.038 mmol, 95%CI: -0.07 to − 0.006), sodium (MD = − 53.285 mmol, 95%CI: -85.823 to − 20.748), and chloride (MD = − 55.809 mmol, 95%CI: -89.035 to − 22.583). Increased urinary excretions of citrate (MD = 0.455 mmol, 95%CI: 0.076–0.835) and magnesium (MD = 0.697 mmol, 95%CI: 0.244–1.149) were found in the low HDL group.

**Conclusions:**

The present study first investigated the effects of dyslipidemia on 24-h urinalysis in NF adults. Of note, high LDL and low HDL were found to be adversely related to kidney stone formation. However, people with high TG and high TC should be cautious of getting kidney stones.

## Introduction

Nephrolithiasis is becoming increasingly common worldwide [1, 2]. Interestingly, the rate of dyslipidemia is also increasing in developed countries and developing areas such as China [2]. Nephrolithiasis is a metabolic disease, and dyslipidemia has been demonstrated to be an independent risk factor for urinary stone formation and recurrence [3, 4]. The concerning underlying mechanisms were reported to be insulin resistance, inflammation, and oxidative stress [3]. These internal environment disturbances together cause urinary lithogenic changes, including hypercalciuria, hyperuricosuria, hyperoxaluria, and hypocitraturia [3]. Therefore, lipid profiles, which are composed of total cholesterol (TC), triglycerides (TG), high-density lipoprotein (HDL), and low-density lipoprotein (LDL), are supposed to be associated with the levels of physicochemical elements in urine.

Changes in 24-h urine composition are markedly related to urinary stone formation. Moreover, 24-h urine composition analysis is a good approach to detect urine physicochemical elements. In a large cohort with nephrolithiasis, Torricelli and his colleagues found that lipid profiles were significantly associated with urinary composition changes. High TC indicated a higher urinary potassium and calcium, whereas low HDL or high triglyceride levels correlated with an elevated urine sodium, oxalate, and uric acid, as well as a lower urine pH [5]. However, in the setting of a non-stone forming (NF) population, the correlation between dyslipidemia and 24-h urine composition was never reported. To clarify this issue, this study was conducted to evaluate the influence of dyslipidemia on 24-h urine composition in adults without urolithiasis.

## Material and methods

### Study population

From May 2013 to July 2014, our group conducted a nationwide epidemiologic study of urolithiasis among adults aged 18 years and older in China. A total of 584 Chinese adults without urolithiasis in six cities (Shanghai, Chongqing, Harbin, Shaoyang, Lanzhou, and Changzhi) were included in this study. These six cities are located in traditional geographical regions of China (south central, southwest, east, north, northeast, and northwest). This study was approved by the Ethics Committee of the First Affiliated Hospital of Guangzhou Medical University, China. Written informed consent was obtained from all the participants.

**Fig. 1:**
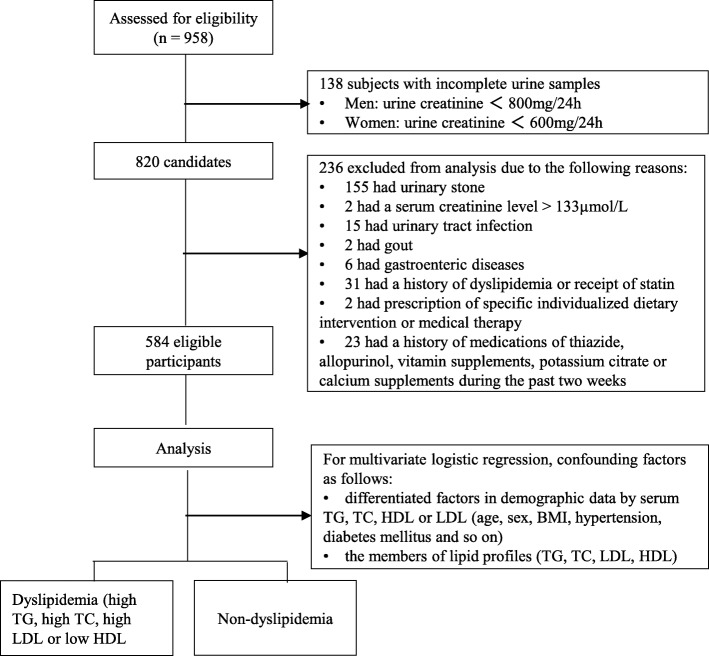
Flow diagram. Subject selection and analysis process

As shown in the Fig. 1, the individuals without nephrolithiasis who underwent at least a single 24-h urine composition analysis and a single fasting lipid profile evaluation were included. Study exclusion criteria were subjects with incomplete urine samples (men: urine creatinine < 800 mg/24 h; women: urine creatinine < 600 mg/24 h), serum creatinine greater than 133 μmol/L, urinary stone, urinary tract infection, gout, gastroenteric diseases, enterectomy history, a history of dyslipidemia or receiving a statin, prescription of specific individualized dietary intervention or medical therapy (except for general fluid or dietary modifications), or a history of taking thiazide, allopurinol, vitamin supplements, potassium citrate, or calcium supplements during the past 2 weeks. All eligible participants with a free diet received ultrasonographic urinary tract examinations and questionnaires to collect the basic characteristic information [e.g., age, gender, body mass index (BMI), health history]. The estimated creatinine clearance rate (eCCr) was calculated by the Cockcroft-Gault equation.

### Collection and analysis of 24-h urine

The detailed methods of the collection and analysis of 24-h urine samples have been described in our previous study [6]. A kit containing all the necessary supplies for the collection was sent to each participant. Clean polyethylene containers with prestored toluene were used to collect urine samples. One 24-h urine sample was collected from each person. All participants maintained their fluid intake and normal diet during the collection.

The urine was analyzed within 72 h after collection. The main measurement outcomes of 24-h urine analysis included urine volume, pH value, and concentration of urine sodium, potassium, chloride, calcium, phosphate, uric acid, magnesium, oxalate, and citrate. Urine citrate and oxalate were measured with ion exchange chromatography (Metrohm, Switzerland). Urine potassium, sodium, calcium, chloride, creatinine, and phosphate were determined by Unicel DxC 600 synchronic biochemical detecting system. Urine uric acid and magnesium were measured by Beckman coulter AU680 automatic biochemistry analyzer. Urine cystine was quantified with Thermo Scientific Microplate Reader. pH values were measured with a glass electrode in a calibrated pH meter (Mettler Toledo, Switzerland). In addition, the ion activity products of calcium oxalate and calcium phosphate, expressed in terms of AP (CaOx) index_s_ and AP (CaP) index_s_ were also calculated in our study [7]. The 24-h urine analyses were performed in the Guangdong Key Laboratory of Urology according to standardized protocols.

$$ AP\ (CaOx)\  indexes=\frac{1.9\ast {\mathrm{Calcium}}^{0.84}\ast \mathrm{Oxalate}}{{\mathrm{Calcium}}^{0.22}\ast {\mathrm{Magnesium}}^{0.12}\ast {1.5}^{1.03}} $$

$$ AP\ (CaP)\  indexes=\frac{2.7\ast {10}^{-3}\ast {\mathrm{Calcium}}^{1.07}\ast {\mathrm{Phospate}}^{0.70}\ast {\left(7.0-4.5\right)}^{6.8}}{{\mathrm{Citrate}}^{0.20}\ast {1.5}^{1.31}} $$

### Measurement and definition of variables

Fasting blood samples were taken from patients to measure their plasma lipid profiles. The samples from different cities were measured with the same analyzer and reagent kits. According to the National Cholesterol Education Program (Adult Treatment Panel III guidelines), dyslipidemia was defined as follows: hypercholesterolemia (TC > 200 mg/dL), hypertriglyceridemia (TG > 150 mg/dL), low HDL cholesterolemia (HDL-C < 40 mg/dL in men and < 50 mg/dL in women), or high-LDL cholesterolemia (LDL cholesterol > 130 mg/dL).

### Statistical analysis

Serum lipid levels were analyzed as dichotomous (dyslipidemia vs normal) to evaluate their effect on 24-h urine composition. The baseline characteristics and 24-h urine composition of the dyslipidemia group (any abnormality in the lipid profile: hypercholesterolemia, hypertriglyceridemia, low HDL-C or high LDL-C) were compared with their corresponding normal group, respectively. Student’s t-test was used on continuous variables and chi-squared for categorical variables. Univariable comparisons of urinary components between dyslipidemia group and normal group were conducted with Student’s t-test. The multivariate logistic regression was performed to access the independent impact of all possible factors in baseline characteristics comparisons between dyslipidemia group and normal group. A two-sided *P* value < 0.05 was considered significant for all results in this study. All statistical analyses were conducted by the SPSS 17.0 software.

## Results

The demographic data and 24-h urine composition of the included adults are listed in Table 1. A total of 584 NF adults (47.6% men) was included. The mean age was 49.98 ± 15.76 years, and the mean BMI was 23.72 ± 3.77. There were 148 (25.3%), 122 (20.9%), and 41 (7%) adults with a history of hypertension, diabetes mellitus, and urolithiasis, respectively.

**Table 1:** Demographic data and 24-h urine composition in 584 adults

Age (Mean ± SD, yrs)	49.98 ± 15.76
BMI (Mean ± SD, kg/m2)	23.72 ± 3.77
Gender, Male (n [%])	278 (47.6%)
Hypertension (n [%])	148 (25.3%)
Diabetes mellitus (n [%])	122 (20.9%)
Urolithiasis history (n [%])	41 (7%)
24-h urinalysis (Mean ± SD)	
Volume (ml)	1459.17 ± 625.46
pH	6.26 ± 0.65
Calcium (mmol)	4.17 ± 2.21
Oxalate (mmol)	0.26 ± 0.15
Citrate (mmol)	2.15 ± 1.38
Uric acid (mmol)	3.14 ± 1.12
Sodium (mmol)	186.02 ± 148.55
Potassium (mmol)	42.91 ± 18.66
Magnesium (mmol)	3.63 ± 1.63
Phosphate (mmol)	17.07 ± 6.51
Creatinine (mmol)	9.95 ± 3.19
Chloride (mmol)	182.13 ± 151.92
AP (CaOx) Index_s_	0.80 ± 0.61
AP (CaP) Index_s_	25.24 ± 18.81

As shown in Table 2, high TG (> 1.7 mmol/L) was more frequently seen in older (53.82 vs. 49.12, *p* = 0.001) and higher BMI (25.30 vs. 23.37, *p* < 0.001) adults. High TG participants also had a higher proportion of individuals with hypertension (37.7% vs. 22.6%, *p* = 0.001) and diabetes mellitus (34% vs. 18%, *p* < 0.001). The participants in the high TC group (> 5.17 mmol/L) were older than those on the normal group (56.34 vs. 47.25, *p* < 0.001). The high TC group had a higher proportion of individuals with hypertension (34.3% vs. 21.5%, *p* = 0.001), diabetes mellitus (30.9% vs. 16.6%, *p* < 0.001), and a history of urolithiasis (11.4% vs. 5.1%, *p* = 0.006). The serum levels of eCCr, calcium, and uric acid were increased in the high TC group. In the high LDL group (> 3.12 mmol/L), the individuals had an older mean age (55.58 ± 15.46 vs. 48.09 ± 15.43, *p* < 0.001) and a higher BMI (25.30 ± 3.26 vs. 23.37 ± 3.78, *p* < 0.001). The participants with high LDL were more likely to have hypertension (36.1% vs. 21.7%, *p* = 0.001) and diabetes mellitus (34% vs. 16.5%, *p* < 0.001). The participants with low HDL (< 0.91 mmol/L) had a younger mean age (39.56 ± 13.34 vs. 51.15 ± 15.59, *p* < 0.001) and a higher BMI (25.45 ± 5.16 vs. 23.53 ± 3.53, *p* = 0.007).

**Table 2:** Demographic data by serum TG, TC, HDL and LDL

Variables	Hypertriglyceridemia (1.7 mmol/L)		*p* Value	Hypercholesterolemia (5.17 mmol/L)		*p* Value
	No (*N* = 478)	Yes (*N* = 106)		No (*N* = 409)	Yes (*N* = 175)	
Age (year)	49.12 ± 16.33	53.82 ± 12.20	0.001	47.25 ± 15.89	56.34 ± 13.48	< 0.001
BMI (kg/m2)	23.37 ± 3.78	25.30 ± 3.26	< 0.001	23.68 ± 3.92	23.81 ± 3.39	0.706
Gender, Male (n [%])	239 (50%)	39 (36.8%)	0.14	196 (47.9%)	82 (46.9%)	0.813
Hypertension (n [%])	108 (22.6%)	40 (37.7%)	0.001	88 (21.5%)	60 (34.3%)	0.001
Diabetes mellitus (n [%])	86 (18%)	36 (34%)	< 0.001	68 (16.6%)	54 (30.9%)	< 0.001
Urolithiasis history (n [%])	35 (7.3%)	6 (5.7%)	0.545	21 (5.1%)	20 (11.4%)	0.006
Hyperuricemia (n [%])	33 (6.9%)	9 (7.2%)	0.567	26 (6.4%)	16 (9.1%)	0.233
Hemoglobin (g/L)	139.72 ± 17.04	139.06 ± 16.23	0.715	139.06 ± 17.55	140.86 ± 15.19	0.239
Serum creatinine (μmol/L)	73.35 ± 17.14	69.91 ± 16.70	0.061	73.34 ± 17.42	71.26 ± 16.28	0.178
eCCr (ml/min)	89.33 ± 30.52	93.67 ± 30.56	0.187	92.48 ± 30.88	84.60 ± 29.10	0.004
Serum calcium (mmol/L)	2.38 ± 0.13	2.41 ± 0.17	0.119	2.37 ± 0.14	2.42 ± 0.13	< 0.001
Serum sodium (mmol/L)	140.25 ± 2.77	140.05 ± 2.44	0.504	140.25 ± 2.91	140.13 ± 2.20	0.574
Serum potassium (mmol/L)	4.14 ± 0.49	4.18 ± 0.43	0.427	4.15 ± 0.49	4.14 ± 0.44	0.765
Serum uric acid (μmol/L)	290.69 ± 82.70	307.67 ± 84.59	0.057	285.71 ± 82.23	312.61 ± 82.74	< 0.001
Variables	LDL-C (3.12 mmol/L)		*p* Value	HDL-C (0.91 mmol/L)		*p* Value
	No (*N* = 437)	Yes (*N* = 147)		No (*N* = 525)	Yes (*N* = 59)	
Age (year)	48.09 ± 15.43	55.58 ± 15.46	< 0.001	51.15 ± 15.59	39.56 ± 13.34	< 0.001
BMI (kg/m2)	23.37 ± 3.78	25.30 ± 3.26	< 0.001	23.53 ± 3.53	25.45 ± 5.16	0.007
Gender, Male (n [%])	212 (48.5%)	66 (44.9%)	0.448	244 (46.4%)	34 (58.6%)	0.077
Hypertension (n [%])	95 (21.7%)	53 (36.1%)	0.001	139 (26.4%)	9 (15.5%)	0.07
Diabetes mellitus (n [%])	72 (16.5%)	50 (34%)	< 0.001	114 (21.7%)	8 (13.8%)	0.161
Urolithiasis history (n [%])	27 (6.2%)	14 (9.5%)	0.17	39 (7.4%)	2 (3.4%)	0.262
Hyperuricemia (n [%])	26 (5.9%)	16 (10.9%)	0.45	37 (7.0%)	5 (8.6%)	0.657
Hemoglobin (g/L)	139.72 ± 17.04	139.06 ± 16.23	0.715	139.62 ± 16.09	139.46 ± 22.95	0.959
Serum creatinine (μmol/L)	73.35 ± 17.14	69.91 ± 16.70	0.061	72.51 ± 16.99	74.63 ± 18.10	0.366
eCCr (ml/min)	89.33 ± 30.52	93.67 ± 30.56	0.187	87.70 ± 27.76	111.67 ± 43.49	< 0.001
Serum calcium (mmol/L)	2.38 ± 0.13	2.41 ± 0.17	0.119	2.39 ± 0.14	2.35 ± 0.12	0.056
Serum sodium (mmol/L)	140.25 ± 2.77	140.05 ± 2.44	0.504	140.14 ± 2.66	140.83 ± 3.14	0.109
Serum potassium (mmol/L)	4.14 ± 0.49	4.18 ± 0.43	0.427	4.14 ± 0.48	4.23 ± 0.40	0.142
Serum uric acid (μmol/L)	290.69 ± 82.70	307.67 ± 84.59	0.057	293.68 ± 82.20	294.58 ± 92.63	0.937

Table 3 shows the influences of dyslipidemic status on 24-h urinalysis data. In the high TG group, the volume of urine was greater than that of the normal group (1594.53 ± 742.61 vs. 1429.15 ± 593.10, *p* = 0.034). The levels of oxalate (0.29 ± 0.13 vs. 0.25 ± 0.15, *p* = 0.026), potassium (47.70 ± 18.66 vs. 41.84 ± 18.51, *p* = 0.003), and AP (CaOx) index (0.92 ± 0.65 vs. 0.78 ± 0.59, *p* = 0.029) were higher in the high TG group. In the high TC group, the individuals were more likely to have lower levels of magnesium (3.44 ± 1.45 vs. 3.72 ± 1.69, *p* = 0.049). The high LDL group had lower levels of sodium (162.68 ± 107.75 vs. 193.87 ± 159.29, *p* = 0.028), magnesium (3.34 ± 1.47 vs. 3.74 ± 1.67, *p* = 0.011), chloride (158.93 ± 108.24 vs. 189.94 ± 163.39, *p* = 0.032), and AP (CaOx) index (0.70 ± 0.46 vs. 0.84 ± 0.64, *p* = 0.004) than the normal group. Interestingly, citrate and magnesium in the urine are inhibitors for renal stone formation. However, our study showed that the levels of 24-h citrate (2.61 ± 1.66 vs. 2.10 ± 1.34, *p* = 0.007) and magnesium (4.53 ± 1.75 vs. 3.53 ± 1.58, *p* < 0.001) in the urine were increased in the low HDL group.

**Table 3:** The influence of dyslipidemic status on 24-h urinalysis data

Variables	Hypertriglyceridemia (1.7 mmol/L)		*p* Value	Hypercholesterolemia (5.17 mmol/L)		*p* Value
	No (*N* = 478)	Yes (*N* = 106)		No (*N* = 409)	Yes (*N* = 175)	
Volume (ml)	1429.15 ± 593.10	1594.53 ± 742.61	0.034	1468.00 ± 623.56	1438.51 ± 631.19	0.602
pH	6.26 ± 0.63	6.27 ± 0.74	0.876	6.27 ± 0.63	6.22 ± 0.69	0.399
Calcium (mmol)	4.12 ± 2.11	4.42 ± 2.61	0.258	4.14 ± 2.23	4.25 ± 2.16	0.593
Oxalate (mmol)	0.25 ± 0.15	0.29 ± 0.13	0.026	0.26 ± 0.15	0.26 ± 0.14	0.699
Citrate (mmol)	2.14 ± 1.43	2.24 ± 1.15	0.496	2.18 ± 1.41	2.09 ± 1.31	0.487
Uric acid (mmol)	3.13 ± 1.10	3.19 ± 1.18	0.632	3.12 ± 1.14	3.18 ± 1.07	0.55
Sodium (mmol)	186.23 ± 159.77	185.07 ± 81.04	0.942	181.74 ± 99.14	196.03 ± 225.27	0.692
Potassium (mmol)	41.84 ± 18.51	47.70 ± 18.66	0.003	43.11 ± 18.76	42.44 ± 18.48	0.692
Magnesium (mmol)	3.62 ± 1.63	3.72 ± 1.63	0.554	3.72 ± 1.69	3.44 ± 1.45	0.049
Phosphate (mmol)	16.86 ± 6.60	18.01 ± 6.03	0.101	16.77 ± 6.74	17.76 ± 5.90	0.093
Creatinine (mmol)	9.96 ± 3.23	9.88 ± 2.98	0.817	9.92 ± 3.28	10.01 ± 2.98	0.755
Chloride (mmol)	181.92 ± 163.71	183.12 ± 80.00	0.941	176.84 ± 99.39	194.50 ± 232.27	0.334
AP (CaOx) Index_s_	0.78 ± 0.59	0.92 ± 0.65	0.029	0.79 ± 0.59	0.85 ± 0.63	0.255
AP (CaP) Index_s_	24.88 ± 18.52	26.87 ± 20.07	0.326	24.82 ± 18.98	26.24 ± 18.42	0.403
Variables	LDL-C (3.12 mmol/L)		*p* Value	HDL-C (0.91 mmol/L)		*p* Value
	No (*N* = 437)	Yes (*N* = 147)		No (*N* = 525)	Yes (*N* = 59)	
Volume (ml)	1452.02 ± 634.94	1480.41 ± 598.01	0.634	1440.06 ± 612.64	1629.15 ± 713.22	0.028
pH	6.24 ± 0.63	6.31 ± 0.71	0.28	6.27 ± 0.66	6.19 ± 0.52	0.397
Calcium (mmol)	4.27 ± 2.24	3.87 ± 2.07	0.053	4.12 ± 2.17	4.68 ± 2.48	0.069
Oxalate (mmol)	0.27 ± 0.15	0.24 ± 0.12	0.075	0.26 ± 0.15	0.26 ± 0.14	0.729
Citrate (mmol)	2.17 ± 1.40	2.10 ± 1.33	0.6	2.10 ± 1.34	2.61 ± 1.66	0.007
Uric acid (mmol)	3.13 ± 1.17	3.16 ± 0.94	0.816	3.11 ± 1.12	3.40 ± 1.08	0.058
Sodium (mmol)	193.87 ± 159.29	162.68 ± 107.75	0.028	184.47 ± 154.56	199.85 ± 75.91	0.451
Potassium (mmol)	42.92 ± 18.18	42.86 ± 20.10	0.97	42.65 ± 18.75	45.16 ± 17.81	0.328
Magnesium (mmol)	3.74 ± 1.67	3.34 ± 1.47	0.011	3.53 ± 1.58	4.53 ± 1.75	< 0.001
Phosphate (mmol)	16.87 ± 6.79	17.66 ± 5.58	0.2	17.04 ± 6.54	17.30 ± 6.31	0.772
Creatinine (mmol)	10.00 ± 3.20	9.79 ± 3.15	0.48	9.91 ± 3.19	10.26 ± 3.21	0.43
Chloride (mmol)	189.94 ± 163.39	158.93 ± 108.24	0.032	180.15 ± 157.98	199.84 ± 78.55	0.346
AP (CaOx) Index_s_	0.84 ± 0.64	0.70 ± 0.46	0.004	0.80 ± 0.60	0.84 ± 0.66	0.636
AP (CaP) Index_s_	25.94 ± 19.89	23.16 ± 15.01	0.076	24.93 ± 18.36	28.07 ± 22.45	0.224

Then, multivariate logistic regression was performed to assess the independent impact of dyslipidemic status on 24-h urine composition considering the confounding factors including age, sex, BMI, hypertension, and diabetes mellitus. Moreover, the members of the lipid profiles were also considered to be confounding factors. For example, if there were differences in terms of the levels of TC, LDL, or HDL between the high TG and normal groups, the status of TC, LDL, or HDL would be considered as a confounding factor when evaluating the independent impact of TG on 24-h urine composition. As shown in Table 4, the high TG status had an independent impact on the increased urinary volume and urinary excretion of oxalate, potassium, and AP (CaOx) index. The high TC status had an independent influence on the increased urinary excretion of calcium, sodium, chloride, AP (CaOx) index, and AP (CaP) index. Of note, the high LDL status had an independent effect on the decreased urinary excretion of calcium, oxalate, sodium, chloride, AP (CaOx) index, and AP (CaP) index. The low HDL status had an independent impact on the increased urinary excretion of citrate and magnesium, as well as decreased urinary excretion of creatinine.

**Table 4:** Multivariate adjusted differences in 24-h urinary excretion between dyslipidemia and normal adults

	Hypertriglyceridemia (1.7 mmol/L)		*p* Value	Hypercholesterolemia (5.17 mmol/L)		*p* Value
	Difference	95%CI		Difference	95%CI	
Volume (ml)	188.361	(49.294, 327.429)	0.008	−77.317	(− 213.049, 58.416)	0.264
pH	0.013	(−0.132, 0.158)	0.861	−0.091	(− 0.232, 0.051)	0.208
Calcium (mmol)	0.337	(−0.146, 0.821)	0.171	0.531	(0.061, 1.001)	0.027
Oxalate (mmol)	0.032	(0.000, 0.065)	0.05	0.014	(−0.017, 0.045)	0.379
Citrate (mmol)	−0.103	(− 0.402, 0.196)	0.498	0.071	(−0.216, 0.358)	0.627
Uric acid (mmol)	0.048	(−0.195, 0.291)	0.697	0.176	(−0.058, 0.411)	0.14
Sodium (mmol)	−11.21	(−44.326, 21.906)	0.506	41.561	(9.179, 73.942)	0.012
Potassium (mmol)	4.298	(0.182, 8.414)	0.041	−2.398	(−6.416, 1.620)	0.242
Magnesium (mmol)	0.103	(−0.254,0.461)	0.571	0.004	(−0.342, 0.349)	0.984
Phosphate (mmol)	0.703	(−0.72, 2.125)	0.332	0.615	(−0.76, 1.991)	0.38
Creatinine (mmol)	0.293	(−0.293, 0.879)	0.326	0.475	(−0.087, 1.037)	0.097
Chloride (mmol)	−9.534	(−43.371, 24.303)	0.58	45.209	(12.118, 78.299)	0.007
AP (CaOx) Index_s_	0.146	(0.012, 0.279)	0.032	0.155	(0.025, 0.284)	0.019
AP (CaP) Index_s_	2.418	(−1.666, 6.502)	0.245	4.2	(0.21, 8.189)	0.039
	LDL-C (3.12 mmol/L)		*p* Value	HDL-C (0.91 mmol/L)		*p* Value
	Difference	95%CI		Difference	95%CI	
Volume (ml)	38.165	(−82.456, 158.787)	0.445	127.322	(−49.633, 304.276)	0.158
pH	0.123	(−0.02, 0.265)	0.092	−0.082	(− 0.267, 0.103)	0.384
Calcium (mmol)	−0.573	(−1.048, − 0.097)	0.018	0.161	(−0.455, 0.778)	0.607
Oxalate (mmol)	−0.038	(−0.07, − 0.006)	0.019	−0.249	(− 0.046, 0.036)	0.804
Citrate (mmol)	−0.093	(−0.387, 0.201)	0.534	0.455	(0.076, 0.835)	0.019
Uric acid (mmol)	0.017	(−0.221, 0.256)	0.886	0.065	(−0.245, 0.374)	0.681
Sodium (mmol)	−53.285	(−-85.823, −20.748)	0.001	15.46	(−26.733, 57.653)	0.472
Potassium (mmol)	−0.706	(−4.758, 3.347)	0.732	1.942	(−3.303, 7.186)	0.467
Magnesium (mmol)	−0.033	(−0.68, 0.021)	0.066	0.697	(0.244, 1.149)	0.003
Phosphate (mmol)	0.329	(−1.083, 1.742)	0.647	−0.819	(−2.612, 0.973)	0.37
Creatinine (mmol)	−0.057	(−0.632, 0.519)	0.847	−0.904	(−1.647, − 0.161)	0.017
Chloride (mmol)	−55.809	(−89.035, −22.583)	0.001	23.066	(−20.023, 66.155)	0.294
AP (CaOx) Index_s_	−0.237	(−0.368, − 0.106)	< 0.001	−0.053	(− 0.223, 0.117)	0.543
AP (CaP) Index_s_	−4.236	(−8.258, −0.214)	0.039	−0.363	(−5.569, 4.843)	0.891

Confounding factors including age, sex, BMI, hypertension, diabetes mellitus and so on according to results of demographic data by serum TG, TC, HDL and LDL. Moreover, the members of lipid profiles were also considered as confounding factors. For example, if there were differences in terms of the levels of TC, LDL or HDL between high TG and normal group, the status of TC, LDL or HDL would be considered as a confounding factor when evaluating the independent impact of TG on 24-h urine composition

## Discussion

The pathophysiology of nephrolithiasis is multifactorial. The components of metabolic syndrome (MetS) including obesity, hypertension, dyslipidemia, and diabetes were risk factors for stone formation [8–11]. Dyslipidemia has been widely accepted to be an independent risk factor for renal stone formation and recurrence [4, 12]. Insulin resistance, inflammation, and oxidative stress were hypothesized to be the mechanism. These internal environment disturbances together cause urinary lithogenic changes, including hypercalciuria, hyperuricosuria, hyperoxaluria, and hypocitraturia [3]. Analysis with 24-h urine composition is a good approach to detect urine physicochemical elements. In the SF patients, dyslipidemia could cause significant changes in the 24-h urine composition [5]. However, in the setting of the NF population, the correlation between dyslipidemia and 24-h urine composition was never reported. Our present study included 584 NF participants from a nationwide epidemiologic study in six cities and for the first time reported the effects of dyslipidemia on 24-h urine composition in adults without urolithiasis. Specifically, high TG and TC caused increased urinary excretion of lithogenic components, such as oxalate, calcium, potassium, sodium, chloride, AP (CaOx) index, and AP (CaP) index. Interestingly, high LDL decreased the urinary excretion of lithogenic components such as calcium, sodium, chloride, AP (CaOx) index, and AP (CaP) index. Low HDL increased the urinary excretion of protective factors for stone formation including citrate and magnesium.

High TG was reported to cause high urinary calcium, sodium, uric acid, magnesium, and potassium excretions in SF patients [4, 5]. In the present study, we showed that high TG had higher urinary oxalate, potassium, and AP (CaOx) index in the NF population. The new finding in this study was that high TG caused high urinary oxalate excretion. Table 2 shows that the patients with increased TG had higher BMI (*P* < 0.001), which suggested that increased oxalate accumulation might be caused by obesity. Recently, Khashayar Sakhaee uncovered the mechanisms of obesity-induced hyperoxaluria. The potential pathophysiologic mechanisms of obesity-induced hyperoxaluria might be as follows [13–16]: (a) increased intake of oxalate and increased oxalate intestinal absorption daily; (b) obese subjects had decreased number of *Oxalobacter formigenes*, which could degrade intestinal luminal oxalate and stimulate intestinal oxalate secretion; (c) increased intestinal and circulating proinflammatory cytokines in obese subjects inhibits intestinal oxalate secretion mediated by the anion exchange transporter Slc26a6 (A6). These potential mechanisms could explain why increased TG cause increased oxalate accumulation. SF patients with high TG level were shown to more likely to have stone recurrence and had a shorter stone recurrence-free period [4]. Andrew J et al. followed the participants without urolithiasis and found by multivariable analysis that the median TG levels of people who formed kidney stones were higher than that of people who did not form kidney stones [17]. Our results implied that NF populations with high TG might form kidney stones more readily due to more urinary oxalate and potassium excretion.

The univariable analysis results demonstrated that high TC caused increased urinary magnesium excretion. However, multivariate logistic regression analysis did not show a significant difference in terms of urinary magnesium. Instead, multivariate logistic regression analysis showed that high TC could cause increased excretion of urinary calcium, sodium, chloride, AP (CaOx) indexes, and AP (CaP) Indexes. High TC was reported to be correlated with high urinary calcium in SF patients [5]. This relation was also suggested in animal studies [18]. The present study first reported that high TC had a link to high urinary sodium, chloride, AP (CaOx) indexes, and AP (CaP) indexes. The present results also demonstrate that people with high TC should pay attention to kidney stone formation.

Therefore, for the people with high TG and high TC, they should be cautious of getting kidney stones. Statin intake and regular exercise had a protective effect against stone formation [19]. Individual components of serum lipids (TC, TG, LDL cholesterol, HDL cholesterol) were reported to be adversely associated with urinary stone risk. Kang et al. found that in SF patients, high TG and low HDL were associated with increased risk for urinary stones, whereas high TC and high LDL were associated with reduced risk of urinary stones [20]. In the NF population, our results also indicated that contrary to the lithogenic urinary changes of high TG and high TC, high LDL caused decreased lithogenic component excretion including calcium, oxalate, sodium, chloride, AP (CaOx) indexes, AP (CaP) indexes, and low HDL caused increased stone formation inhibitors such as citrate and magnesium. Our findings concerning the effects of LDL on 24-h urinary components were similar to the previous studies. High LDL seemed to lower the risk of stones [20]. However, the effects of HDL on 24-h urinary components was different from the existing findings. HDL was shown to have protective anti-inflammatory effects and protect against insulin resistance [21]. Low levels of HDL were reported to increase the nephrolithiasis risk [20, 22]. Moreover, the underlying physiologic mechanism explaining why individual components of dyslipidemia have a contradictory effect on lithogenic urinary changes cannot be explained at present. For the population without urolithiasis, the latest study including the participants with a first recorded diagnosis of hyperlipidemia also got a conflicting result. During the follow-up, they found that the participants who developed kidney stones had a lower level TC or LDL and higher HDL. However, they confirmed that higher TG was associated with an increased risk of kidney stones [17].

Some limitations in this present study should be stated. Because the 24-h urine samples were collected from a NF population in a nationwide epidemiology study of urolithiasis, the dietary habits of the participants could not be controlled, and they differed much due to distant remote locations. Different diets may potentially influence the 24-h urine composition, and this bias could not be adjusted in the multivariate analysis. Furthermore, the climate and environment are also different among the included cities, and the numbers of people with dyslipidemia in our study are relatively small. All these factors may cause potential bias to our results.

## Conclusion

The present study for the first time investigated the effects of dyslipidemia on 24-h urinalysis in adults without urolithiasis. Of note, high LDL and low HDL were found to be adversely related to kidney stone formation. However, according to our results, people with high TG and high TC should be cautious of getting kidney stones.

## Abbreviations

BMI: Body mass index
CI: Confidence interval
eCCr: Creatinine clearance rate
HDL: High density lipoprotein
LDL: Low-density lipoprotein
MD: Mean difference
NF: Non-stone formers
TC: Total cholesterol
TG: Triglycerides
